# 2-((*E*)-{2-[(1*E*)-(2,4-Dihydroxy­benzyl­idene)amino]phen­yl}iminiometh­yl)-5-hydroxy­phenolate methanol solvate

**DOI:** 10.1107/S1600536808014487

**Published:** 2008-06-13

**Authors:** Naser Eltaher Eltayeb, Siang Guan Teoh, Suchada Chantrapromma, Hoong-Kun Fun, Rohana Adnan

**Affiliations:** a School of Chemical Science, Universiti Sains Malaysia, 11800 USM, Penang, Malaysia; b Department of Chemistry, Faculty of Science, Prince of Songkla University, Hat-Yai, Songkhla 90112, Thailand; c X-ray Crystallography Unit, School of Physics, Universiti Sains Malaysia, 11800 USM, Penang, Malaysia

## Abstract

The asymmetric unit of the title compound, C_20_H_16_N_2_O_4_·CH_3_OH, contains two Schiff base zwitterions and two methanol solvent mol­ecules. The dihedral angles between the central benzene ring and the two outer benzene rings of the Schiff base are 2.57 (7) and 52.30 (7)° in one mol­ecule and 5.83 (7) and 49.82 (7)° in the other mol­ecule. Intra­molecular O—H⋯N and N—H⋯O hydrogen bonds generate *S*(6) ring motifs, whereas intra­molecular N—H⋯N hydrogen bonds generate *S*(5) ring motifs. In the crystal structure, O—H⋯O, hydrogen bonds and weak C—H⋯O inter­actions link the mol­ecules into one-dimensional chains along the *b*-axis direction and are further connected by O—H⋯O and weak C—H⋯O inter­actions into a three-dimensional network. C—H⋯π and π–π inter­actions [centroid–centroid distances = 3.6228 (9) and 3.6881 (9) Å] are also observed in the crystal structure.

## Related literature

For bond-length data, see: Allen *et al.* (1987 ▶). For details of hydrogen-bond motifs, see: Bernstein *et al.* (1995 ▶). For related structures, see, for example: Eltayeb *et al.* (2007*a* ▶,*b* ▶). For background to applications of Schiff base ligands, see, for example: Dao *et al.* (2000 ▶); Eltayeb & Ahmed (2005*a* ▶,*b* ▶); Fakhari *et al.* (2005 ▶); Karthikeyan *et al.* (2006 ▶); Sriram *et al.* (2006 ▶).
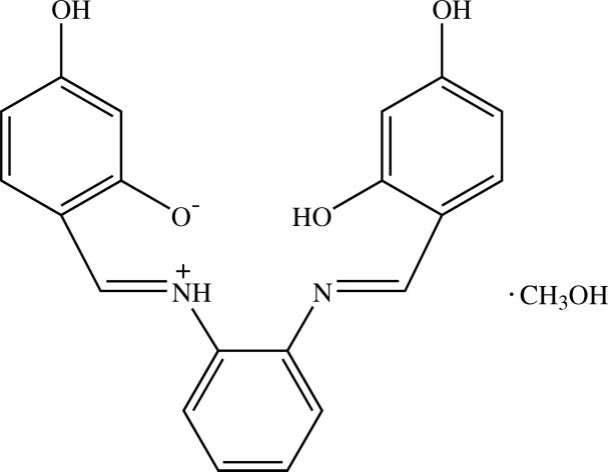


## Experimental

### 

#### Crystal data

C_20_H_16_N_2_O_4_·CH_4_O
*M* *_r_* = 380.39
Triclinic, 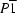

*a* = 8.3672 (2) Å
*b* = 11.0813 (2) Å
*c* = 20.3217 (3) Å
α = 89.313 (1)°
β = 80.309 (1)°
γ = 79.641 (1)°
*V* = 1826.73 (6) Å^3^
*Z* = 4
Mo *K*α radiation
μ = 0.10 mm^−1^
*T* = 100.0 (1) K
0.50 × 0.34 × 0.17 mm

#### Data collection

Bruker SMART APEX2 CCD area-detector diffractometer
Absorption correction: multi-scan (*SADABS*; Bruker, 2005 ▶) *T*_min_ = 0.952, *T*_max_ = 0.984
42593 measured reflections
10651 independent reflections
7203 reflections with *I* > 2σ(*I*)’
*R*_int_ = 0.034

#### Refinement

*R*[*F* ^2^ > 2σ(*F* ^2^)] = 0.056
*wR*(*F* ^2^) = 0.162
*S* = 1.05
10651 reflections
515 parameters
H-atom parameters constrained
Δρ_max_ = 0.44 e Å^−3^
Δρ_min_ = −0.34 e Å^−3^

### 

Data collection: *APEX2* (Bruker, 2005 ▶); cell refinement: *APEX2*; data reduction: *SAINT* (Bruker, 2005 ▶); program(s) used to solve structure: *SHELXTL* (Sheldrick, 2008 ▶); program(s) used to refine structure: *SHELXTL*; molecular graphics: *SHELXTL*; software used to prepare material for publication: *SHELXTL* and *PLATON* (Spek, 2003 ▶).

## Supplementary Material

Crystal structure: contains datablocks global, I. DOI: 10.1107/S1600536808014487/sj2499sup1.cif

Structure factors: contains datablocks I. DOI: 10.1107/S1600536808014487/sj2499Isup2.hkl

Additional supplementary materials:  crystallographic information; 3D view; checkCIF report

## Figures and Tables

**Table 1:** Hydrogen-bond geometry (Å, °)

*D*—H⋯*A*	*D*—H	H⋯*A*	*D*⋯*A*	*D*—H⋯*A*
O1*A*—H1*OA*⋯O5*B*^i^	0.95	1.71	2.6610 (16)	176
O3*A*—H3*OA*⋯N2*A*	0.96	1.78	2.6637 (16)	153
O4*A*—H4*OA*⋯O2*A*^ii^	0.82	1.83	2.6330 (16)	164
N1*A*—H1*NA*⋯O2*A*	0.92	1.84	2.6021 (16)	138
N1*A*—H1*NA*⋯N2*A*	0.92	2.31	2.7063 (16)	106
O1*B*—H1*OB*⋯O5*A*^iii^	0.99	1.64	2.6205 (16)	170
O3*B*—H3*OB*⋯N2*B*	0.94	1.77	2.6526 (16)	154
O4*B*—H4*OB*⋯O2*B*^iv^	0.89	1.74	2.6241 (16)	174
N1*B*—H1*NB*⋯O2*B*	0.87	1.88	2.6006 (16)	139
N1*B*—H1*NB*⋯N2*B*	0.87	2.32	2.7020 (16)	107
O5*A*—H5*OA*⋯O2*B*	0.84	1.91	2.7145 (16)	162
O5*A*—H5*OA*⋯O3*B*	0.84	2.58	2.9703 (15)	110
O5*B*—H5*OB*⋯O2*A*	0.91	1.83	2.7034 (16)	160
C4*A*—H4*A*⋯O5*B*^i^	0.93	2.48	3.165 (2)	131
C4*B*—H4*B*⋯O5*A*^iii^	0.93	2.48	3.1596 (19)	130
C7*A*—H7*A*⋯O4*B*^iv^	0.93	2.36	3.1691 (17)	146
C7*B*—H7*B*⋯O4*A*^ii^	0.93	2.35	3.1253 (17)	141
C12*B*—H12*B*⋯O1*B*^v^	0.93	2.55	3.3603 (18)	146
C21*B*—H21*D*⋯O3*A*	0.96	2.44	3.134 (2)	129
C21*B*—H21*D*⋯*Cg*3^vi^	0.96	2.86	3.568 (2)	132

Symmetry codes: (i) 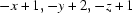; (ii) 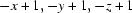; (iii) 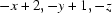; (iv) 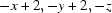; (v) 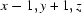; (vi) 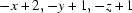. *Cg*3 is the centroid of the C15*A*–C20*A* ring.

**Fig. 1.**
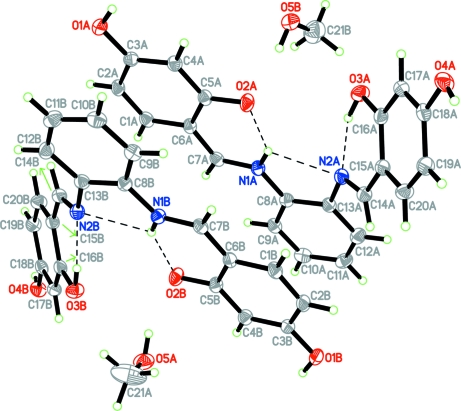
The asymmetric unit of (I), showing 50% probability displacement ellipsoids and the atomic numbering. Intramolecular O—H···N, N—H···O and N—H···N hydrogen bonds are drawn as dashed lines.

**Fig. 2.**
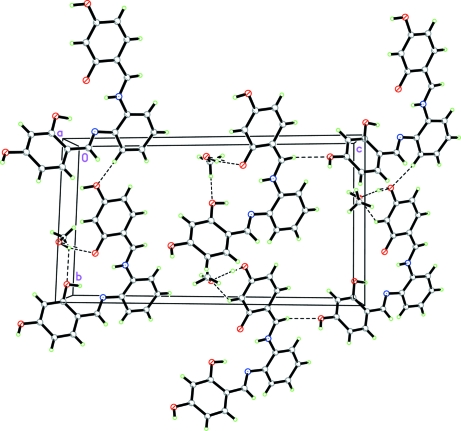
The crystal packing of (I), viewed along the a axis, showing the molecular chains along the b axis. Hydrogen bonds are drawn as dashed lines.

## supplementary crystallographic information

### Comment

Schiff bases have received much attention because of their potential applications with some of these compounds exhibiting various pharmacological activities, as noted by their anticancer (Dao *et al.*, 2000), anti-HIV (Sriram *et al.*, 2006), antibacterial and antifungal (Karthikeyan *et al.*, 2006) properties. In addition, some of them may be used as analytical reagents for the determination of trace elements (Eltayeb & Ahmed, 2005*a*,*b*) such as nickel in some natural food products (Fakhari *et al.*, 2005). We reported the crystal structures of 5,5'-Dimethoxy-2,2'-[1,2-phenylenebis(nitrilomethylidyne)diphenol (Eltayeb *et al.*,  2007*a*) and 4,4'-Dimethoxy-2,2'-[1,2-phenylenebis(nitrilomethylidyne)diphenol (Eltayeb *et al.*,  2007*b*) and we report here the structure of the title compound (I), a closely-related Schiff base.

The asymmetric unit of (I) (Fig. 1) contains two Schiff base zwitterions and two methanol molecules (*A* and *B*). The zwitterion results from protonation of the imine N1A and N1B atoms with  protons from the O2A and O2B hydroxy groups  resulting in the formation of iminium and hydroxyphenolate groups. In the structure, the hydroxyphenolate ring (C1–C6/O1-O2) is nearly coplanar with the phenyl ring (C8–C13) as indicated by the dihedral angles between these two rings being 2.57 (7)° in molecule *A* and 5.83 (7)° in molecule *B* and the torsion angle C8/N1/C7/C6 = 179.33 (2)° in molecule *A* and 178.07 (12)° in molecule *B*. The C8–C13 phenyl ring makes a dihedral angle of 52.30 (7)° with the dihydroxyphenyl ring (C15–C20/O3–O4) in molecule *A* [49.82 (7)° in molecule *B*].

Intramolecular hydrogen bonds, O3A—H3OA···N2A, N1A—H1NA···O2A, O3B—H3OB···N2B and N1B—H1NB···O2B (Table 1) generate S(6) ring motifs whereas N1A—H1NA···N2A and N1B—H1NB···N2B generate S(5) ring motifs (Bernstein *et al.*, 1995). Bond lengths and angles are in normal ranges (Allen *et al.*,  1987) and comparable to those in related structures (Eltayeb *et al.*, 2007*a,b*). In the crystal packing (Fig. 2), O—H···O, hydrogen bonds and weak C—H···O interactions (Table 1) link the molecules into one dimensional chains along the *b* direction and are further connected by O—H···O and weak C—H···O interactions (Table 1) into a three-dimensional network (Table 1). The crystal is further stabilized by weak C—H···π interactions (Table 1). π···π interactions were also observed with the distances of *Cg*_1_···*Cg*_5_ = 3.6228 (9) Å  and *Cg*_2_···*Cg*_4_ = 3.6881 (9) Å (symmetry code : x, y, z in each case);  *Cg*_1_, *Cg*_2_, *Cg*_3_, *Cg*_4_ and *Cg*_5_ are the centroids of the C1A–C6A, C8A–C13A, C15A–C20A, C1B–C6B and C8B–C13B benzene rings, respectively.

### Experimental

The title compound was synthesized by adding 2,4-dihydroxybenzaldehyde (0.552 g, 4 mmol) to a solution of *o*-phenylenediamine (0.216 g, 2 mmol) in ethanol (20 ml). The mixture was refluxed with stirring for half an hour. The resultant yellow solution was filtered. Yellow single crystals of the title compound suitable for *x*-ray structure determination were recrystallized from ethanol by slow evaporation of the solvent at room temperature over several days.

### Refinement

Hydroxyl and imine H atoms were located from the difference map and refined riding on the parent atoms with refinement of the isotropic thermal parameters. The remaining H atoms were placed in calculated positions with d(C—H) = 0.93 Å, *U*_iso_=1.2*U*_eq_(C) for aromatic, CH, 0.96 Å, *U*_iso_ = 1.5*U*_eq_(C) for CH_3_ atoms A rotating group model was used for the methyl groups.

### Crystal data

**Table:**

C_20_H_16_N_2_O_4_·CH_4_O	*Z* = 4
*M**_r_* = 380.39	*F*_000_ = 800
Triclinic, *P*1	*D*_x_ = 1.383 Mg m^−^^3^
Hall symbol: -P 1	Mo *K*α radiation λ = 0.71073 Å
*a* = 8.3672 (2) Å	Cell parameters from 10651 reflections
*b* = 11.0813 (2) Å	θ = 1.0–30.0º
*c* = 20.3217 (3) Å	µ = 0.10 mm^−^^1^
α = 89.313 (1)º	*T* = 100.0 (1) K
β = 80.309 (1)º	Block, yellow
γ = 79.641 (1)º	0.50 × 0.34 × 0.17 mm
*V* = 1826.73 (6) Å^3^	

### Data collection

**Table:**

Bruker SMART APEX2 CCD area-detector diffractometer	10651 independent reflections
Radiation source: fine-focus sealed tube	7203 reflections with *I* > 2σ(*I*)'
Monochromator: graphite	*R*_int_ = 0.034
Detector resolution: 8.33 pixels mm^-1^	θ_max_ = 30.0º
*T* = 100.0(1) K	θ_min_ = 1.0º
ω scans	*h* = −11→10
Absorption correction: multi-scan(SADABS; Bruker, 2005)	*k* = −15→15
*T*_min_ = 0.952, *T*_max_ = 0.984	*l* = −28→28
42593 measured reflections	

### Refinement

**Table:**

Refinement on *F*^2^	Secondary atom site location: difference Fourier map
Least-squares matrix: full	Hydrogen site location: inferred from neighbouring sites
*R*[*F*^2^ > 2σ(*F*^2^)] = 0.056	H-atom parameters constrained
*wR*(*F*^2^) = 0.162	*w* = 1/[σ^2^(*F*_o_^2^) + (0.0788*P*)^2^ + 0.3402*P*] where *P* = (*F*_o_^2^ + 2*F*_c_^2^)/3
*S* = 1.05	(Δ/σ)_max_ < 0.001
10651 reflections	Δρ_max_ = 0.44 e Å^−^^3^
515 parameters	Δρ_min_ = −0.34 e Å^−^^3^
Primary atom site location: structure-invariant direct methods	Extinction correction: none

### Special details

**Table:**

Experimental. The low-temperature data was collected with the Oxford Cyrosystem Cobra low-temperature attachment.
Geometry. All esds (except the esd in the dihedral angle between two l.s. planes) are estimated using the full covariance matrix. The cell esds are taken into account individually in the estimation of esds in distances, angles and torsion angles; correlations between esds in cell parameters are only used when they are defined by crystal symmetry. An approximate (isotropic) treatment of cell esds is used for estimating esds involving l.s. planes.
Refinement. Refinement of F^2^ against ALL reflections. The weighted R-factor wR and goodness of fit S are based on F^2^, conventional R-factors R are based on F, with F set to zero for negative F^2^. The threshold expression of F^2^ > 2sigma(F^2^) is used only for calculating R-factors(gt) etc. and is not relevant to the choice of reflections for refinement. R-factors based on F^2^ are statistically about twice as large as those based on F, and R- factors based on ALL data will be even larger.

### Fractional atomic coordinates and isotropic or equivalent isotropic displacement parameters (Å^2^)

**Table:**

	*x*	*y*	*z*	*U*_iso_*/*U*_eq_	
O1A	0.38240 (14)	1.21218 (9)	0.37963 (5)	0.0269 (3)	
H1OA	0.3231	1.1862	0.4197	0.055 (6)*	
O2A	0.70037 (13)	0.81358 (9)	0.39164 (5)	0.0243 (2)	
O3A	0.83682 (14)	0.59449 (9)	0.48780 (5)	0.0268 (3)	
H3OA	0.8948	0.5957	0.4431	0.054 (6)*	
O4A	0.49475 (14)	0.34820 (10)	0.60844 (5)	0.0272 (3)	
H4OA	0.4485	0.2903	0.6031	0.061 (7)*	
N1A	0.94132 (15)	0.74067 (10)	0.29384 (6)	0.0193 (3)	
H1NA	0.8739	0.7270	0.3329	0.042 (5)*	
N2A	0.97260 (16)	0.52908 (11)	0.36228 (6)	0.0211 (3)	
C1A	0.73415 (19)	1.05359 (13)	0.26751 (7)	0.0220 (3)	
H1A	0.7991	1.0678	0.2273	0.026*	
C2A	0.60758 (19)	1.14282 (13)	0.29478 (7)	0.0231 (3)	
H2A	0.5870	1.2175	0.2736	0.028*	
C3A	0.50713 (19)	1.12139 (12)	0.35571 (7)	0.0215 (3)	
C4A	0.53930 (19)	1.01151 (12)	0.38853 (7)	0.0213 (3)	
H4A	0.4740	0.9996	0.4290	0.026*	
C5A	0.66928 (18)	0.91814 (12)	0.36127 (7)	0.0201 (3)	
C6A	0.76932 (18)	0.93925 (12)	0.29904 (7)	0.0191 (3)	
C7A	0.90059 (18)	0.84951 (12)	0.26841 (7)	0.0198 (3)	
H7A	0.9618	0.8679	0.2282	0.024*	
C8A	1.06914 (18)	0.64458 (12)	0.26672 (7)	0.0191 (3)	
C9A	1.17540 (19)	0.65455 (13)	0.20737 (7)	0.0220 (3)	
H9A	1.1653	0.7274	0.1840	0.026*	
C10A	1.29635 (19)	0.55566 (14)	0.18314 (8)	0.0251 (3)	
H10A	1.3668	0.5620	0.1432	0.030*	
C11A	1.31301 (19)	0.44739 (14)	0.21809 (8)	0.0260 (3)	
H11A	1.3952	0.3815	0.2017	0.031*	
C12A	1.20798 (19)	0.43678 (13)	0.27732 (8)	0.0250 (3)	
H12A	1.2205	0.3640	0.3007	0.030*	
C13A	1.08334 (18)	0.53462 (13)	0.30223 (7)	0.0204 (3)	
C14A	0.90878 (19)	0.43132 (13)	0.37372 (7)	0.0221 (3)	
H14A	0.9346	0.3700	0.3408	0.026*	
C15A	0.80086 (19)	0.41174 (12)	0.43391 (7)	0.0206 (3)	
C16A	0.76986 (19)	0.49236 (12)	0.49003 (7)	0.0206 (3)	
C17A	0.67009 (19)	0.46828 (13)	0.54807 (7)	0.0223 (3)	
H17A	0.6535	0.5202	0.5851	0.027*	
C18A	0.59436 (19)	0.36593 (13)	0.55096 (7)	0.0220 (3)	
C19A	0.6219 (2)	0.28485 (13)	0.49599 (7)	0.0243 (3)	
H19A	0.5723	0.2159	0.4983	0.029*	
C20A	0.72290 (19)	0.30880 (13)	0.43892 (7)	0.0241 (3)	
H20A	0.7405	0.2555	0.4024	0.029*	
O1B	1.06277 (14)	0.29566 (9)	0.12539 (5)	0.0263 (2)	
H1OB	1.1264	0.3111	0.0813	0.060 (7)*	
O2B	0.78690 (13)	0.70842 (9)	0.11637 (5)	0.0246 (2)	
O3B	0.65725 (14)	0.90440 (9)	0.01575 (5)	0.0263 (2)	
H3OB	0.5866	0.9158	0.0575	0.050 (6)*	
O4B	1.01686 (14)	1.13045 (10)	−0.11108 (5)	0.0269 (2)	
H4OB	1.0777	1.1886	−0.1138	0.065 (7)*	
N1B	0.53881 (15)	0.78591 (10)	0.21044 (6)	0.0188 (3)	
H1NB	0.6050	0.7965	0.1739	0.043 (6)*	
N2B	0.50773 (16)	0.98985 (11)	0.13603 (6)	0.0212 (3)	
C1B	0.72248 (19)	0.46765 (13)	0.23763 (7)	0.0218 (3)	
H1B	0.6540	0.4555	0.2773	0.026*	
C2B	0.84138 (19)	0.37362 (13)	0.21012 (7)	0.0227 (3)	
H2B	0.8535	0.2978	0.2306	0.027*	
C3B	0.94691 (19)	0.39181 (12)	0.14999 (7)	0.0209 (3)	
C4B	0.92878 (19)	0.50368 (13)	0.11862 (7)	0.0218 (3)	
H4B	0.9983	0.5136	0.0789	0.026*	
C5B	0.80678 (18)	0.60236 (12)	0.14599 (7)	0.0195 (3)	
C6B	0.70035 (18)	0.58425 (12)	0.20722 (7)	0.0189 (3)	
C7B	0.57163 (18)	0.67648 (12)	0.23642 (7)	0.0191 (3)	
H7B	0.5062	0.6598	0.2760	0.023*	
C8B	0.40985 (18)	0.88345 (12)	0.23481 (7)	0.0186 (3)	
C9B	0.30337 (19)	0.87881 (13)	0.29475 (7)	0.0223 (3)	
H9B	0.3152	0.8092	0.3208	0.027*	
C10B	0.17954 (19)	0.97810 (14)	0.31555 (8)	0.0249 (3)	
H10B	0.1086	0.9754	0.3558	0.030*	
C11B	0.16100 (19)	1.08150 (14)	0.27664 (8)	0.0264 (3)	
H11B	0.0766	1.1474	0.2906	0.032*	
C12B	0.26700 (19)	1.08732 (13)	0.21731 (8)	0.0245 (3)	
H12B	0.2533	1.1570	0.1914	0.029*	
C13B	0.39474 (19)	0.98924 (13)	0.19586 (7)	0.0204 (3)	
C14B	0.56811 (19)	1.08790 (13)	0.12067 (7)	0.0222 (3)	
H14B	0.5372	1.1544	0.1504	0.027*	
C15B	0.68020 (19)	1.09985 (12)	0.06030 (7)	0.0212 (3)	
C16B	0.72244 (19)	1.00770 (12)	0.00920 (7)	0.0211 (3)	
C17B	0.83261 (19)	1.02095 (13)	−0.04789 (7)	0.0225 (3)	
H17B	0.8579	0.9607	−0.0814	0.027*	
C18B	0.90569 (19)	1.12444 (13)	−0.05529 (7)	0.0222 (3)	
C19B	0.8645 (2)	1.21803 (13)	−0.00595 (7)	0.0253 (3)	
H19B	0.9114	1.2883	−0.0115	0.030*	
C20B	0.7541 (2)	1.20413 (13)	0.05044 (7)	0.0255 (3)	
H20B	0.7275	1.2658	0.0832	0.031*	
O5A	0.78442 (15)	0.63854 (10)	−0.01097 (6)	0.0355 (3)	
H5OA	0.7819	0.6756	0.0250	0.053*	
C21A	0.6263 (3)	0.6108 (3)	−0.00516 (12)	0.0693 (8)	
H21A	0.5458	0.6836	0.0074	0.104*	
H21B	0.6128	0.5498	0.0283	0.104*	
H21C	0.6116	0.5797	−0.0472	0.104*	
O5B	0.77865 (16)	0.87056 (10)	0.50980 (6)	0.0370 (3)	
H5OB	0.7381	0.8399	0.4761	0.055*	
C21B	0.9533 (3)	0.84547 (17)	0.50003 (11)	0.0475 (5)	
H21D	0.9915	0.7584	0.4966	0.071*	
H21E	0.9903	0.8780	0.5372	0.071*	
H21F	0.9964	0.8831	0.4597	0.071*	

### Atomic displacement parameters (Å^2^)

**Table:**

	*U*^11^	*U*^22^	*U*^33^	*U*^12^	*U*^13^	*U*^23^
O1A	0.0347 (7)	0.0192 (5)	0.0245 (6)	0.0013 (5)	−0.0056 (5)	0.0008 (4)
O2A	0.0273 (6)	0.0196 (5)	0.0238 (5)	−0.0020 (4)	−0.0007 (5)	0.0079 (4)
O3A	0.0374 (7)	0.0182 (5)	0.0266 (6)	−0.0113 (5)	−0.0032 (5)	−0.0006 (4)
O4A	0.0351 (7)	0.0275 (6)	0.0202 (5)	−0.0114 (5)	−0.0025 (5)	0.0051 (4)
N1A	0.0202 (7)	0.0189 (6)	0.0191 (6)	−0.0048 (5)	−0.0029 (5)	0.0028 (4)
N2A	0.0232 (7)	0.0197 (6)	0.0207 (6)	−0.0045 (5)	−0.0039 (5)	0.0032 (5)
C1A	0.0274 (8)	0.0213 (7)	0.0196 (7)	−0.0087 (6)	−0.0065 (6)	0.0048 (5)
C2A	0.0318 (9)	0.0162 (6)	0.0238 (7)	−0.0063 (6)	−0.0101 (7)	0.0057 (5)
C3A	0.0253 (8)	0.0174 (6)	0.0233 (7)	−0.0037 (6)	−0.0082 (6)	−0.0016 (5)
C4A	0.0250 (8)	0.0200 (7)	0.0193 (7)	−0.0052 (6)	−0.0033 (6)	0.0028 (5)
C5A	0.0227 (8)	0.0182 (6)	0.0214 (7)	−0.0067 (6)	−0.0063 (6)	0.0039 (5)
C6A	0.0202 (8)	0.0182 (6)	0.0209 (7)	−0.0061 (6)	−0.0067 (6)	0.0034 (5)
C7A	0.0225 (8)	0.0217 (7)	0.0177 (7)	−0.0086 (6)	−0.0054 (6)	0.0030 (5)
C8A	0.0173 (7)	0.0204 (6)	0.0204 (7)	−0.0048 (5)	−0.0042 (6)	−0.0001 (5)
C9A	0.0233 (8)	0.0238 (7)	0.0207 (7)	−0.0075 (6)	−0.0060 (6)	0.0028 (5)
C10A	0.0214 (8)	0.0304 (8)	0.0236 (7)	−0.0080 (6)	−0.0007 (6)	−0.0029 (6)
C11A	0.0201 (8)	0.0247 (7)	0.0321 (8)	−0.0020 (6)	−0.0029 (7)	−0.0064 (6)
C12A	0.0246 (8)	0.0210 (7)	0.0299 (8)	−0.0041 (6)	−0.0061 (7)	−0.0003 (6)
C13A	0.0227 (8)	0.0206 (7)	0.0194 (7)	−0.0061 (6)	−0.0054 (6)	0.0018 (5)
C14A	0.0253 (8)	0.0198 (6)	0.0222 (7)	−0.0046 (6)	−0.0065 (6)	0.0012 (5)
C15A	0.0244 (8)	0.0181 (6)	0.0200 (7)	−0.0053 (6)	−0.0043 (6)	0.0023 (5)
C16A	0.0239 (8)	0.0169 (6)	0.0228 (7)	−0.0051 (6)	−0.0076 (6)	0.0036 (5)
C17A	0.0284 (9)	0.0194 (6)	0.0202 (7)	−0.0041 (6)	−0.0069 (6)	0.0008 (5)
C18A	0.0244 (8)	0.0218 (7)	0.0198 (7)	−0.0037 (6)	−0.0049 (6)	0.0056 (5)
C19A	0.0290 (9)	0.0210 (7)	0.0246 (8)	−0.0096 (6)	−0.0042 (6)	0.0033 (6)
C20A	0.0292 (9)	0.0208 (7)	0.0235 (7)	−0.0077 (6)	−0.0038 (6)	−0.0017 (6)
O1B	0.0326 (7)	0.0182 (5)	0.0253 (6)	0.0023 (4)	−0.0047 (5)	0.0008 (4)
O2B	0.0270 (6)	0.0182 (5)	0.0257 (5)	−0.0024 (4)	0.0011 (5)	0.0069 (4)
O3B	0.0342 (7)	0.0198 (5)	0.0261 (6)	−0.0106 (5)	−0.0024 (5)	−0.0001 (4)
O4B	0.0331 (7)	0.0283 (6)	0.0203 (5)	−0.0107 (5)	−0.0024 (5)	0.0052 (4)
N1B	0.0196 (6)	0.0179 (5)	0.0188 (6)	−0.0040 (5)	−0.0019 (5)	0.0013 (4)
N2B	0.0234 (7)	0.0198 (6)	0.0206 (6)	−0.0041 (5)	−0.0040 (5)	0.0029 (5)
C1B	0.0254 (8)	0.0227 (7)	0.0191 (7)	−0.0080 (6)	−0.0049 (6)	0.0060 (5)
C2B	0.0290 (9)	0.0174 (6)	0.0234 (7)	−0.0065 (6)	−0.0072 (6)	0.0059 (5)
C3B	0.0241 (8)	0.0187 (6)	0.0209 (7)	−0.0030 (6)	−0.0077 (6)	0.0000 (5)
C4B	0.0235 (8)	0.0208 (7)	0.0203 (7)	−0.0038 (6)	−0.0018 (6)	0.0029 (5)
C5B	0.0209 (8)	0.0178 (6)	0.0210 (7)	−0.0061 (6)	−0.0042 (6)	0.0043 (5)
C6B	0.0196 (8)	0.0185 (6)	0.0201 (7)	−0.0051 (6)	−0.0059 (6)	0.0027 (5)
C7B	0.0208 (8)	0.0219 (7)	0.0163 (6)	−0.0076 (6)	−0.0040 (6)	0.0031 (5)
C8B	0.0170 (7)	0.0192 (6)	0.0207 (7)	−0.0042 (5)	−0.0048 (6)	−0.0014 (5)
C9B	0.0227 (8)	0.0240 (7)	0.0214 (7)	−0.0079 (6)	−0.0034 (6)	0.0012 (5)
C10B	0.0211 (8)	0.0316 (8)	0.0222 (7)	−0.0079 (6)	−0.0004 (6)	−0.0036 (6)
C11B	0.0196 (8)	0.0255 (7)	0.0335 (8)	−0.0029 (6)	−0.0036 (7)	−0.0063 (6)
C12B	0.0261 (8)	0.0194 (7)	0.0293 (8)	−0.0042 (6)	−0.0080 (7)	0.0013 (6)
C13B	0.0218 (8)	0.0202 (6)	0.0207 (7)	−0.0056 (6)	−0.0057 (6)	−0.0003 (5)
C14B	0.0262 (8)	0.0197 (7)	0.0213 (7)	−0.0041 (6)	−0.0063 (6)	0.0003 (5)
C15B	0.0255 (8)	0.0188 (6)	0.0206 (7)	−0.0048 (6)	−0.0074 (6)	0.0024 (5)
C16B	0.0252 (8)	0.0184 (6)	0.0222 (7)	−0.0060 (6)	−0.0091 (6)	0.0042 (5)
C17B	0.0280 (9)	0.0200 (7)	0.0202 (7)	−0.0042 (6)	−0.0063 (6)	0.0004 (5)
C18B	0.0246 (8)	0.0244 (7)	0.0187 (7)	−0.0059 (6)	−0.0062 (6)	0.0066 (5)
C19B	0.0315 (9)	0.0204 (7)	0.0262 (8)	−0.0095 (6)	−0.0061 (7)	0.0037 (6)
C20B	0.0323 (9)	0.0221 (7)	0.0234 (8)	−0.0085 (6)	−0.0045 (7)	0.0005 (6)
O5A	0.0446 (8)	0.0320 (6)	0.0259 (6)	−0.0036 (5)	0.0020 (5)	−0.0061 (5)
C21A	0.0710 (18)	0.0941 (19)	0.0535 (14)	−0.0471 (15)	−0.0052 (12)	−0.0218 (13)
O5B	0.0476 (8)	0.0312 (6)	0.0264 (6)	0.0059 (6)	−0.0035 (6)	−0.0034 (5)
C21B	0.0521 (14)	0.0349 (10)	0.0598 (13)	−0.0127 (9)	−0.0163 (11)	0.0007 (9)

### Geometric parameters (Å, °)

**Table:**

O1A—C3A	1.3420 (17)	O4B—C18B	1.3493 (18)
O1A—H1OA	0.9500	O4B—H4OB	0.8863
O2A—C5A	1.3113 (16)	N1B—C7B	1.3183 (17)
O3A—C16A	1.3473 (16)	N1B—C8B	1.4112 (18)
O3A—H3OA	0.9560	N1B—H1NB	0.8691
O4A—C18A	1.3503 (18)	N2B—C14B	1.2921 (18)
O4A—H4OA	0.8240	N2B—C13B	1.4097 (19)
N1A—C7A	1.3169 (17)	C1B—C2B	1.357 (2)
N1A—C8A	1.4098 (18)	C1B—C6B	1.4209 (18)
N1A—H1NA	0.9215	C1B—H1B	0.9300
N2A—C14A	1.2934 (18)	C2B—C3B	1.417 (2)
N2A—C13A	1.4105 (19)	C2B—H2B	0.9300
C1A—C2A	1.358 (2)	C3B—C4B	1.3828 (19)
C1A—C6A	1.4186 (19)	C4B—C5B	1.403 (2)
C1A—H1A	0.9300	C4B—H4B	0.9300
C2A—C3A	1.418 (2)	C5B—C6B	1.438 (2)
C2A—H2A	0.9300	C6B—C7B	1.400 (2)
C3A—C4A	1.3868 (19)	C7B—H7B	0.9300
C4A—C5A	1.402 (2)	C8B—C9B	1.389 (2)
C4A—H4A	0.9300	C8B—C13B	1.4053 (19)
C5A—C6A	1.436 (2)	C9B—C10B	1.384 (2)
C6A—C7A	1.405 (2)	C9B—H9B	0.9300
C7A—H7A	0.9300	C10B—C11B	1.385 (2)
C8A—C9A	1.389 (2)	C10B—H10B	0.9300
C8A—C13A	1.4054 (19)	C11B—C12B	1.380 (2)
C9A—C10A	1.383 (2)	C11B—H11B	0.9300
C9A—H9A	0.9300	C12B—C13B	1.397 (2)
C10A—C11A	1.384 (2)	C12B—H12B	0.9300
C10A—H10A	0.9300	C14B—C15B	1.434 (2)
C11A—C12A	1.383 (2)	C14B—H14B	0.9300
C11A—H11A	0.9300	C15B—C20B	1.4028 (19)
C12A—C13A	1.396 (2)	C15B—C16B	1.4206 (19)
C12A—H12A	0.9300	C16B—C17B	1.379 (2)
C14A—C15A	1.432 (2)	C17B—C18B	1.389 (2)
C14A—H14A	0.9300	C17B—H17B	0.9300
C15A—C20A	1.4069 (19)	C18B—C19B	1.407 (2)
C15A—C16A	1.4169 (19)	C19B—C20B	1.371 (2)
C16A—C17A	1.378 (2)	C19B—H19B	0.9300
C17A—C18A	1.391 (2)	C20B—H20B	0.9300
C17A—H17A	0.9300	O5A—C21A	1.398 (2)
C18A—C19A	1.404 (2)	O5A—H5OA	0.8380
C19A—C20A	1.368 (2)	C21A—H21A	0.9600
C19A—H19A	0.9300	C21A—H21B	0.9600
C20A—H20A	0.9300	C21A—H21C	0.9600
O1B—C3B	1.3428 (17)	O5B—C21B	1.418 (2)
O1B—H1OB	0.9923	O5B—H5OB	0.9079
O2B—C5B	1.3106 (16)	C21B—H21D	0.9600
O3B—C16B	1.3498 (16)	C21B—H21E	0.9600
O3B—H3OB	0.9451	C21B—H21F	0.9600
			
C3A—O1A—H1OA	108.9	C2B—C1B—C6B	121.45 (14)
C16A—O3A—H3OA	104.7	C2B—C1B—H1B	119.3
C18A—O4A—H4OA	109.0	C6B—C1B—H1B	119.3
C7A—N1A—C8A	127.48 (13)	C1B—C2B—C3B	119.54 (13)
C7A—N1A—H1NA	114.2	C1B—C2B—H2B	120.2
C8A—N1A—H1NA	118.2	C3B—C2B—H2B	120.2
C14A—N2A—C13A	118.47 (12)	O1B—C3B—C4B	122.39 (14)
C2A—C1A—C6A	121.38 (14)	O1B—C3B—C2B	116.82 (12)
C2A—C1A—H1A	119.3	C4B—C3B—C2B	120.78 (13)
C6A—C1A—H1A	119.3	C3B—C4B—C5B	120.80 (14)
C1A—C2A—C3A	119.60 (13)	C3B—C4B—H4B	119.6
C1A—C2A—H2A	120.2	C5B—C4B—H4B	119.6
C3A—C2A—H2A	120.2	O2B—C5B—C4B	121.44 (13)
O1A—C3A—C4A	122.46 (14)	O2B—C5B—C6B	120.08 (13)
O1A—C3A—C2A	116.87 (12)	C4B—C5B—C6B	118.46 (12)
C4A—C3A—C2A	120.67 (13)	C7B—C6B—C1B	118.99 (13)
C3A—C4A—C5A	120.66 (14)	C7B—C6B—C5B	122.01 (12)
C3A—C4A—H4A	119.7	C1B—C6B—C5B	118.96 (13)
C5A—C4A—H4A	119.7	N1B—C7B—C6B	123.07 (13)
O2A—C5A—C4A	121.24 (13)	N1B—C7B—H7B	118.5
O2A—C5A—C6A	120.17 (13)	C6B—C7B—H7B	118.5
C4A—C5A—C6A	118.59 (12)	C9B—C8B—C13B	120.41 (13)
C7A—C6A—C1A	119.15 (13)	C9B—C8B—N1B	122.93 (12)
C7A—C6A—C5A	121.77 (12)	C13B—C8B—N1B	116.65 (13)
C1A—C6A—C5A	119.08 (13)	C10B—C9B—C8B	119.76 (14)
N1A—C7A—C6A	123.28 (13)	C10B—C9B—H9B	120.1
N1A—C7A—H7A	118.4	C8B—C9B—H9B	120.1
C6A—C7A—H7A	118.4	C9B—C10B—C11B	120.23 (15)
C9A—C8A—C13A	120.45 (13)	C9B—C10B—H10B	119.9
C9A—C8A—N1A	122.92 (12)	C11B—C10B—H10B	119.9
C13A—C8A—N1A	116.61 (13)	C12B—C11B—C10B	120.40 (14)
C10A—C9A—C8A	119.76 (13)	C12B—C11B—H11B	119.8
C10A—C9A—H9A	120.1	C10B—C11B—H11B	119.8
C8A—C9A—H9A	120.1	C11B—C12B—C13B	120.44 (14)
C9A—C10A—C11A	120.34 (15)	C11B—C12B—H12B	119.8
C9A—C10A—H10A	119.8	C13B—C12B—H12B	119.8
C11A—C10A—H10A	119.8	C12B—C13B—C8B	118.71 (14)
C12A—C11A—C10A	120.27 (14)	C12B—C13B—N2B	123.18 (13)
C12A—C11A—H11A	119.9	C8B—C13B—N2B	118.09 (13)
C10A—C11A—H11A	119.9	N2B—C14B—C15B	123.63 (13)
C11A—C12A—C13A	120.43 (14)	N2B—C14B—H14B	118.2
C11A—C12A—H12A	119.8	C15B—C14B—H14B	118.2
C13A—C12A—H12A	119.8	C20B—C15B—C16B	117.67 (14)
C12A—C13A—C8A	118.72 (14)	C20B—C15B—C14B	120.20 (13)
C12A—C13A—N2A	122.94 (13)	C16B—C15B—C14B	122.12 (13)
C8A—C13A—N2A	118.32 (13)	O3B—C16B—C17B	118.42 (12)
N2A—C14A—C15A	124.04 (13)	O3B—C16B—C15B	120.78 (13)
N2A—C14A—H14A	118.0	C17B—C16B—C15B	120.79 (13)
C15A—C14A—H14A	118.0	C16B—C17B—C18B	119.86 (13)
C20A—C15A—C16A	117.79 (13)	C16B—C17B—H17B	120.1
C20A—C15A—C14A	119.92 (13)	C18B—C17B—H17B	120.1
C16A—C15A—C14A	122.29 (13)	O4B—C18B—C17B	117.28 (13)
O3A—C16A—C17A	118.50 (12)	O4B—C18B—C19B	122.12 (13)
O3A—C16A—C15A	120.74 (13)	C17B—C18B—C19B	120.60 (14)
C17A—C16A—C15A	120.76 (13)	C20B—C19B—C18B	118.98 (13)
C16A—C17A—C18A	119.73 (13)	C20B—C19B—H19B	120.5
C16A—C17A—H17A	120.1	C18B—C19B—H19B	120.5
C18A—C17A—H17A	120.1	C19B—C20B—C15B	122.06 (13)
O4A—C18A—C17A	117.35 (13)	C19B—C20B—H20B	119.0
O4A—C18A—C19A	121.91 (13)	C15B—C20B—H20B	119.0
C17A—C18A—C19A	120.74 (14)	C21A—O5A—H5OA	103.6
C20A—C19A—C18A	119.01 (13)	O5A—C21A—H21A	109.5
C20A—C19A—H19A	120.5	O5A—C21A—H21B	109.5
C18A—C19A—H19A	120.5	H21A—C21A—H21B	109.5
C19A—C20A—C15A	121.93 (13)	O5A—C21A—H21C	109.5
C19A—C20A—H20A	119.0	H21A—C21A—H21C	109.5
C15A—C20A—H20A	119.0	H21B—C21A—H21C	109.5
C3B—O1B—H1OB	113.4	C21B—O5B—H5OB	112.5
C16B—O3B—H3OB	103.5	O5B—C21B—H21D	109.5
C18B—O4B—H4OB	118.9	O5B—C21B—H21E	109.5
C7B—N1B—C8B	127.42 (13)	H21D—C21B—H21E	109.5
C7B—N1B—H1NB	114.3	O5B—C21B—H21F	109.5
C8B—N1B—H1NB	118.3	H21D—C21B—H21F	109.5
C14B—N2B—C13B	118.92 (12)	H21E—C21B—H21F	109.5
			
C6A—C1A—C2A—C3A	−0.6 (2)	C6B—C1B—C2B—C3B	0.4 (2)
C1A—C2A—C3A—O1A	−178.94 (13)	C1B—C2B—C3B—O1B	179.61 (13)
C1A—C2A—C3A—C4A	1.6 (2)	C1B—C2B—C3B—C4B	−0.8 (2)
O1A—C3A—C4A—C5A	178.95 (12)	O1B—C3B—C4B—C5B	−179.67 (12)
C2A—C3A—C4A—C5A	−1.6 (2)	C2B—C3B—C4B—C5B	0.8 (2)
C3A—C4A—C5A—O2A	−179.61 (13)	C3B—C4B—C5B—O2B	−179.15 (13)
C3A—C4A—C5A—C6A	0.6 (2)	C3B—C4B—C5B—C6B	−0.4 (2)
C2A—C1A—C6A—C7A	179.34 (13)	C2B—C1B—C6B—C7B	177.55 (13)
C2A—C1A—C6A—C5A	−0.4 (2)	C2B—C1B—C6B—C5B	0.0 (2)
O2A—C5A—C6A—C7A	0.9 (2)	O2B—C5B—C6B—C7B	1.3 (2)
C4A—C5A—C6A—C7A	−179.35 (13)	C4B—C5B—C6B—C7B	−177.51 (13)
O2A—C5A—C6A—C1A	−179.39 (12)	O2B—C5B—C6B—C1B	178.79 (12)
C4A—C5A—C6A—C1A	0.36 (19)	C4B—C5B—C6B—C1B	0.00 (19)
C8A—N1A—C7A—C6A	179.33 (12)	C8B—N1B—C7B—C6B	178.07 (12)
C1A—C6A—C7A—N1A	−179.54 (13)	C1B—C6B—C7B—N1B	−177.73 (13)
C5A—C6A—C7A—N1A	0.2 (2)	C5B—C6B—C7B—N1B	−0.2 (2)
C7A—N1A—C8A—C9A	1.4 (2)	C7B—N1B—C8B—C9B	4.9 (2)
C7A—N1A—C8A—C13A	−177.32 (13)	C7B—N1B—C8B—C13B	−176.36 (13)
C13A—C8A—C9A—C10A	−0.2 (2)	C13B—C8B—C9B—C10B	1.3 (2)
N1A—C8A—C9A—C10A	−178.88 (12)	N1B—C8B—C9B—C10B	179.98 (13)
C8A—C9A—C10A—C11A	−0.7 (2)	C8B—C9B—C10B—C11B	0.4 (2)
C9A—C10A—C11A—C12A	0.5 (2)	C9B—C10B—C11B—C12B	−0.9 (2)
C10A—C11A—C12A—C13A	0.5 (2)	C10B—C11B—C12B—C13B	−0.2 (2)
C11A—C12A—C13A—C8A	−1.3 (2)	C11B—C12B—C13B—C8B	1.9 (2)
C11A—C12A—C13A—N2A	179.96 (13)	C11B—C12B—C13B—N2B	−179.56 (13)
C9A—C8A—C13A—C12A	1.2 (2)	C9B—C8B—C13B—C12B	−2.4 (2)
N1A—C8A—C13A—C12A	179.95 (12)	N1B—C8B—C13B—C12B	178.80 (12)
C9A—C8A—C13A—N2A	179.95 (12)	C9B—C8B—C13B—N2B	178.95 (12)
N1A—C8A—C13A—N2A	−1.28 (18)	N1B—C8B—C13B—N2B	0.20 (18)
C14A—N2A—C13A—C12A	−43.96 (19)	C14B—N2B—C13B—C12B	43.0 (2)
C14A—N2A—C13A—C8A	137.33 (14)	C14B—N2B—C13B—C8B	−138.49 (14)
C13A—N2A—C14A—C15A	177.04 (13)	C13B—N2B—C14B—C15B	−178.21 (13)
N2A—C14A—C15A—C20A	172.92 (14)	N2B—C14B—C15B—C20B	−173.14 (14)
N2A—C14A—C15A—C16A	−7.8 (2)	N2B—C14B—C15B—C16B	5.9 (2)
C20A—C15A—C16A—O3A	−178.04 (13)	C20B—C15B—C16B—O3B	179.28 (13)
C14A—C15A—C16A—O3A	2.7 (2)	C14B—C15B—C16B—O3B	0.2 (2)
C20A—C15A—C16A—C17A	1.7 (2)	C20B—C15B—C16B—C17B	0.1 (2)
C14A—C15A—C16A—C17A	−177.56 (13)	C14B—C15B—C16B—C17B	−178.99 (13)
O3A—C16A—C17A—C18A	177.61 (13)	O3B—C16B—C17B—C18B	−178.07 (13)
C15A—C16A—C17A—C18A	−2.2 (2)	C15B—C16B—C17B—C18B	1.1 (2)
C16A—C17A—C18A—O4A	−178.44 (13)	C16B—C17B—C18B—O4B	177.70 (12)
C16A—C17A—C18A—C19A	1.8 (2)	C16B—C17B—C18B—C19B	−2.0 (2)
O4A—C18A—C19A—C20A	179.29 (13)	O4B—C18B—C19B—C20B	−178.04 (13)
C17A—C18A—C19A—C20A	−0.9 (2)	C17B—C18B—C19B—C20B	1.7 (2)
C18A—C19A—C20A—C15A	0.5 (2)	C18B—C19B—C20B—C15B	−0.4 (2)
C16A—C15A—C20A—C19A	−0.9 (2)	C16B—C15B—C20B—C19B	−0.5 (2)
C14A—C15A—C20A—C19A	178.42 (14)	C14B—C15B—C20B—C19B	178.67 (14)

### Hydrogen-bond geometry (Å, °)

**Table:**

*D*—H···*A*	*D*—H	H···*A*	*D*···*A*	*D*—H···*A*
O1A—H1OA···O5B^i^	0.95	1.71	2.6610 (16)	176
O3A—H3OA···N2A	0.96	1.78	2.6637 (16)	153
O4A—H4OA···O2A^ii^	0.82	1.83	2.6330 (16)	164
N1A—H1NA···O2A	0.92	1.84	2.6021 (16)	138
N1A—H1NA···N2A	0.92	2.31	2.7063 (16)	106
O1B—H1OB···O5A^iii^	0.99	1.64	2.6205 (16)	170
O3B—H3OB···N2B	0.94	1.77	2.6526 (16)	154
O4B—H4OB···O2B^iv^	0.89	1.74	2.6241 (16)	174
N1B—H1NB···O2B	0.87	1.88	2.6006 (16)	139
N1B—H1NB···N2B	0.87	2.32	2.7020 (16)	107
O5A—H5OA···O2B	0.84	1.91	2.7145 (16)	162
O5A—H5OA···O3B	0.84	2.58	2.9703 (15)	110
O5B—H5OB···O2A	0.91	1.83	2.7034 (16)	160
C4A—H4A···O5B^i^	0.93	2.48	3.165 (2)	131
C4B—H4B···O5A^iii^	0.93	2.48	3.1596 (19)	130
C7A—H7A···O4B^iv^	0.93	2.36	3.1691 (17)	146
C7B—H7B···O4A^ii^	0.93	2.35	3.1253 (17)	141
C12B—H12B···O1B^v^	0.93	2.55	3.3603 (18)	146
C21B—H21D···O3A	0.96	2.44	3.134 (2)	129
C21B—H21D···Cg3^vi^	0.96	2.86	3.568 (2)	132

Symmetry codes: (i) −*x*+1, −*y*+2, −*z*+1;  (ii) −*x*+1, −*y*+1, −*z*+1; (iii) −*x*+2, −*y*+1, −*z*; (iv) −*x*+2, −*y*+2, −*z*; (v) *x*−1, *y*+1, *z*; (vi) −*x*+2, −*y*+1, −*z*+1.
